# The Role of Virtual Reality and Functional Electrical Stimulation on a Seven-Year-Old Child With Erb's Palsy: A Case Report

**DOI:** 10.7759/cureus.63393

**Published:** 2024-06-28

**Authors:** Swarna Singh, H. V. Sharath, Gauri Bhutada, Raghumahanti Raghuveer, Nitika Chavan

**Affiliations:** 1 Department of Neurophysiotherapy, Center for Advanced Physiotherapy Education and Research, Ravi Nair Physiotherapy College, Datta Meghe Institute of Higher Education and Research (Deemed to be University), Wardha, IND; 2 Department of Pediatric Physiotherapy, Center for Advanced Physiotherapy Education and Research, Ravi Nair Physiotherapy College, Datta Meghe Institute of Higher Education and Research (Deemed to be University), Wardha, IND; 3 Department of Cardiovascular and Respiratory Physiotherapy, Center for Advanced Physiotherapy Education and Research, Ravi Nair Physiotherapy College, Datta Meghe Institute of Higher Education and Research (Deemed to be University), Wardha, IND

**Keywords:** brachial plexus injury, rehabilitation, pediatrics, functional electrical stimulation, virtual reality, erb's palsy

## Abstract

Erb's palsy usually commonly arises from incidents such as falls, collisions, birth trauma, and shoulder injuries in children. It impairs upper extremity muscle function, which has an impact on the quality of life and social interaction. Physical therapy is beneficial in preserving and enhancing upper extremity function, improving the quality of life. In this case report, a seven-year-old female child with complaints of weakness in the right upper limb demonstrated a notable increase in the strength and function of the upper extremities after four weeks of structured rehabilitation using virtual reality and functional electrical stimulation.

## Introduction

When the brachial plexus (C5, C6), which innervates the arm, is severed, the condition is called Erb's palsy. This leads to an internally rotated shoulder and a pronated forearm, which is sometimes referred to as the "waiter's tip" [1]. The most frequent cause, dystocia (linked to challenging breech and forceps deliveries), can result in partial or complete paralysis depending on the extent of nerve damage, which can range from bruising to ripping. It can also happen after a clavicle fracture unrelated to dystocia or following a traumatic fall at any age [2]. Among children with multiple traumas, 0.1% get brachial plexus damage due to auto accidents or pedestrians hit by cars [3]. The most frequent cause of brachial plexus injuries (BPIs) in children is due to birth injury, with an estimated prevalence ranging from 0.9 to 2.6 per 1,000 live births [4].

Erb's palsy is a clinically diagnosed condition, although certain investigations can also confirm it [5]. Magnetic resonance imaging (MRI) of the cervical spine and brachial plexus is the best method [6]. An X-ray of the shoulder rules out any bone fractures and issues with the elbow and shoulder [7]. The electrical activity of the muscle can be estimated and recorded using electromyography (EMG). When there are no fibrillations on EMG, it suggests neuropraxia. The transit time of an electrical stimulus across a specific nerve can be measured by nerve conduction studies (NCS) [8].

The best course of treatment for Erb's palsy depends on its severity; some cases require surgery, while others can be managed with physiotherapy alone [9]. The treatment approach calls for early immobilization, which is followed by range-of-motion (ROM) exercises [10]. It has been established that a variety of therapy approaches are successful in regaining upper limb function.

Based on context, virtual reality (VR) technology develops three-dimensional virtual environments and allows users to interact with these environments and their elements [11]. With the use of real-time feedback and difficulty levels that can be adjusted to the child's functional level, these systems enable high-intensity, task-oriented motor and sensory training that can support children's needs and increase their desire to participate in the rehabilitation process, with or without matched disabled contemporaries [12].

Functional electrical stimulation (FES) is one of the most promising therapeutic innovations in the field of contemporary clinical rehabilitation. FES is used for the stimulation of motor and sensory neurons, helps in the acceleration of nerve growth, and improves the precise growth of nerve fibers into innervated skeletal muscles along the direction of the electric field. FES is one of the most promising therapeutic innovations in the field of contemporary clinical rehabilitation. It can be used to treat BPIs, aid in regenerating damaged brachial plexus, and stop skeletal muscle denervation atrophy [13].

In this case report, we describe a case of a seven-year-old girl referred to physiotherapy for upper limb weakness.

## Case presentation

A seven-year-old girl had an alleged history of road traffic accidents followed by a fall on her right shoulder. She became unconscious at the time of the accident and was immediately taken to a nearby hospital, after which the patient was taken to a government hospital in Amravati, where she was admitted to the pediatric unit and regained consciousness after two days. Later, the doctor informed the caregiver about the right-side clavicle fracture. The fracture was managed with a shoulder immobilizer for six weeks. After a few months, it was noticeable that the child could not move her right arm, which was ignored by the patient's caregivers as they were not very well aware of the condition. Gradually, it progressed to numbness, muscle atrophy, and muscle weakness in the right arm. After one year, when the child could not lift her arm and there was a marked increase in muscle atrophy and weakness, she was taken to the hospital for management. Further, MRI and nerve conduction velocity were done, suggesting a C5-C6 preganglionic BPI. The child was advised to undergo physiotherapy.

On clinical examination

**Figure 1:**
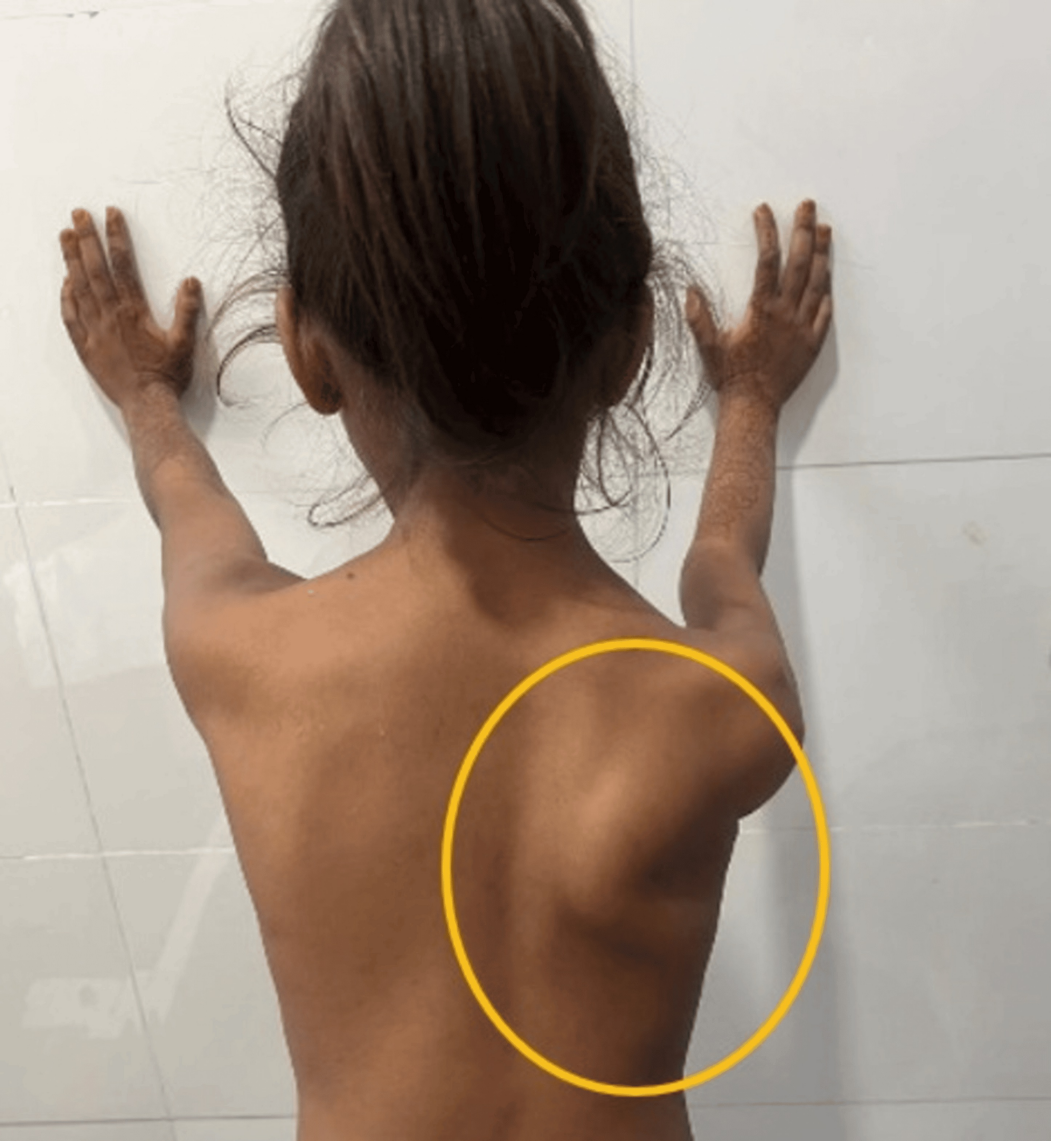
Winging of the scapula in a seven-year-old girl

All dermatomal levels were intact, but there was an impairment in myotomes C5-C6. The active range of motion (AROM) of shoulder flexion and shoulder abduction was 120° and 60°, respectively, while the passive range of motion (PROM) was full. On the right side, muscle circumference was reduced by 2 cm. The wall push-up test showed medial winging of the scapula (Figure 1).

We evaluated all muscle groups supplied by C5-C6 on manual muscle testing, as detailed in Table 1.

**Table 1:** Individual muscle testing Grade 3: full range of motion against gravity; Grade 4: full range of motion against gravity, moderate resistance; Grade 5: full range of motion against gravity, maximal resistance

Muscles	Right	Left
Serratus anterior, rhomboids, and levator scapulae	Grade 4	Grade 5
Deltoid	Grade 3	Grade 5
Teres major, infraspinatus, subscapularis, and supraspinatus	Grade 3	Grade 5
Coracobrachialis, brachialis, and biceps brachii	Grade 3	Grade 5
Teres minor	Grade 3	Grade 5
Elbow flexors	Grade 3	Grade 5
Wrist flexors	Grade 5	Grade 5
Wrist extensor	Grade 5	Grade 5

Nerve conduction study

An NCS suggests that the right brachial plexopathy lesion at the C5-C6 root is mentioned in Tables 2, 3.

**Table 2:** Sensory NCS R: right; L: left; NCS: nerve conduction study

Nerve/sites	Recording site	Onset latency (ms)	Amp 1-2 µV	Segments	Distance (mm)	Velocity (m/s)
R median-digit ll (antidromic)						
Wrist	Index	1.7	35.3	Wrist-index	120	72
L median-digit ll (antidromic)						
Wrist	Index	2	42	Wrist-index	120	61
R ulnar-digit V (antidromic)						
Wrist	Digit V	1.3	35.7	Wrist-digit V	100	77
L ulnar-digit V (antidromic)						
Wrist	Digit V	1.6	40	Wrist-digit V	100	62
R radial-superficial (antidromic)						
Forearm	Wrist	1.7	32.4	Forearm wrist	100	58
L radial-superficial (antidromic)						
Forearm	Wrist	2	23.4	Forearm wrist	100	49

**Table 3:** Motor NCS R: right; L: left; APB: abductor pollicis brevis; ADM: abductor digiti minimi; EIP: extensor indicis proprius; NCS: nerve conduction study

Nerve/sites	Muscles	Latency (ms)	Amplitude (mV)	Segments	Distance (mm)	Latency difference (ms)	Velocity (m/s)
R median-APB							
Wrist	APB	2.67	8.0	Wrist-APB	80	-	-
Elbow	APB	5.48	7.1	Elbow-wrist	170	2.81	60.4
L median-APB							
Wrist	APB	3.25	5.2	Wrist-APB	80	-	-
Elbow	APB	5.81	4.6	Elbow-wrist	170	2.56	66.3
R ulnar-ADM							
Wrist	ADM	1.79	7.9	Wrist-ADM	80	-	-
Below elbow	ADM	4.15	7.0	Below elbow-wrist	190	2.35	80.7
L ulnar-ADM							
Wrist	ADM	2.23	8.1	Wrist-ADM	80	-	-
Below elbow	ADM	4.17	6.8	Below elbow-wrist	190	1.94	98.1
R radial-EIP							
Forearm	EIP	2.00	4.0	Forearm-EIP	-	-	-
Elbow	EIP	4.44	3.9	Elbow-forearm	180	2.44	73.8
L radial-EIP							
Forearm	EIP	1.77	4.2	Forearm-EIP	-	-	-
Elbow	EIP	3.58	3.4	Elbow-forearm	180	1.81	99.3
R axillary-deltoid							
Erb's palsy	Deltoid	2.90	2.1				
L axillary-deltoid							
Erb's palsy	Deltoid	2.48	9.4				
R musculocutaneous-biceps							
Axilla	Deltoid	3.73	1.8				
R musculocutaneous-biceps							
Axilla	Deltoid	3.06	5.3				

Clinical investigation

**Figure 2:**
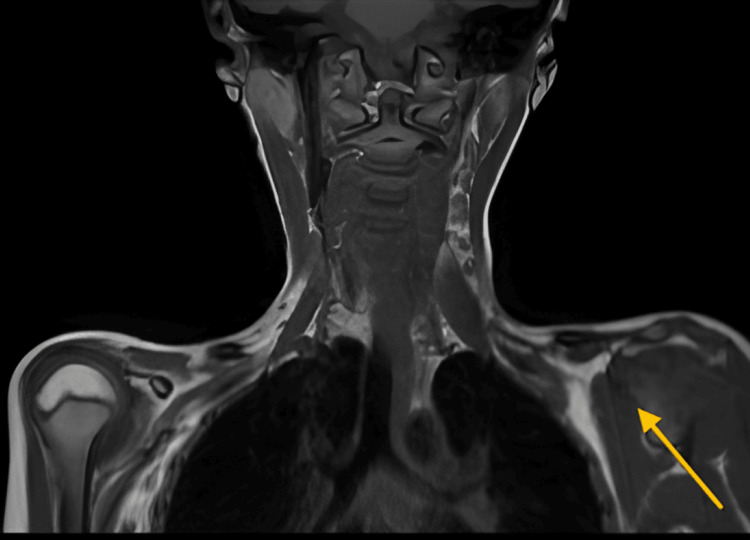
MRI brachial plexus of a seven-year-old girl, suggesting right preganglionic injury (yellow arrow) MRI: magnetic resonance imaging

An MRI report indicating findings suggestive of a right preganglionic injury typically involves the brachial plexus, which is a network of nerves that originates from the spinal cord and supplies the shoulder, arm, and hand, as mentioned in Figure 2.

Physiotherapy intervention

**Figure 3:**
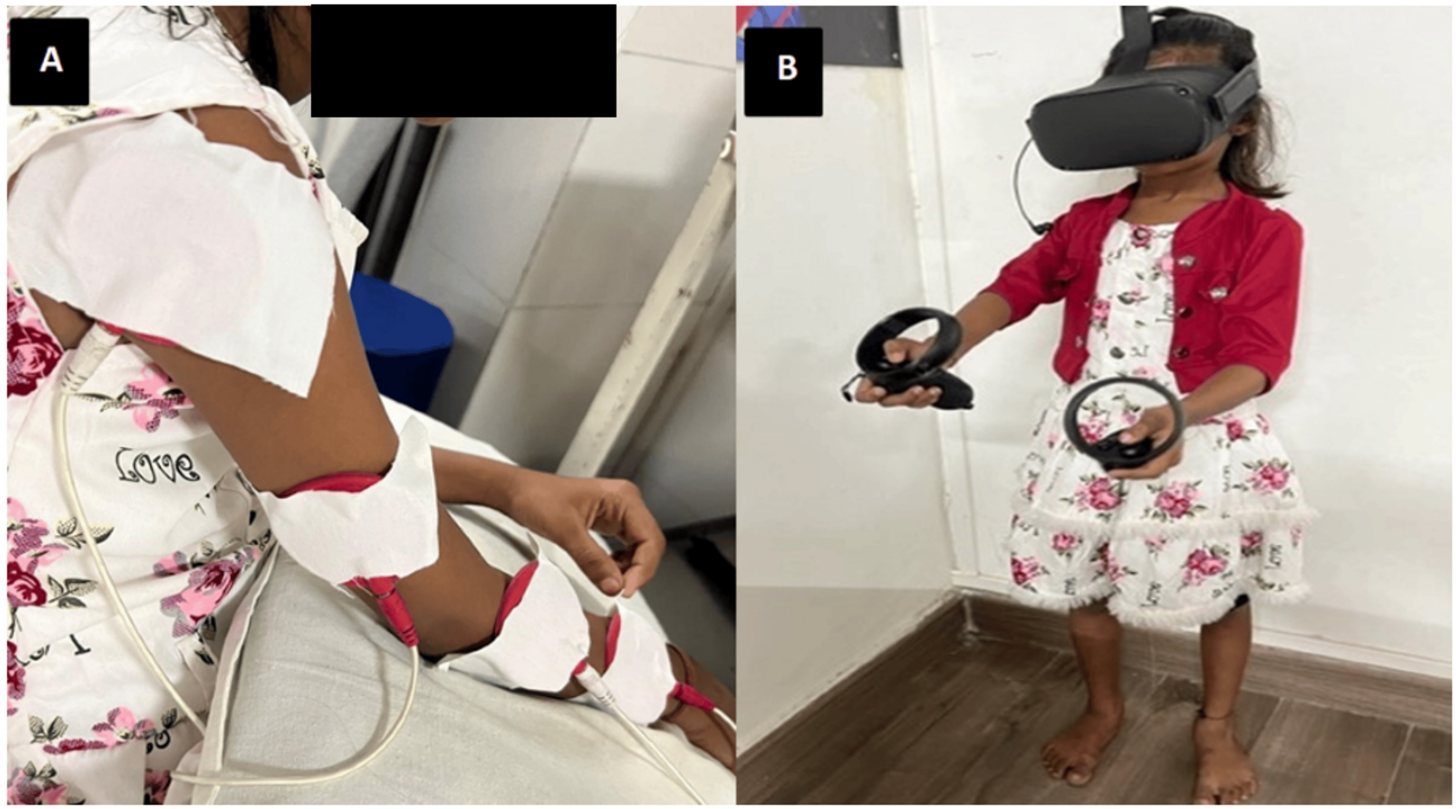
Physiotherapy rehabilitation. (A) FES. (B) VR rehabilitation FES: functional electrical stimulation; VR: virtual reality

The rehabilitation program includes older physiotherapy techniques to improve strength in the affected area. It involves free exercise regimens, strengthening exercises, multiple angles isometric exercises, and brief resisted isometric exercises. The program is conducted for 20 minutes, five days a week, with progression to mechanical and progressive resisted exercises. Improving the utilization of the affected extremities employs modified constraint-induced movement therapy (mCIMT) for six hours a day, divided into two sessions with an hour gap, aiming for an increase in sensorimotor frequency and intensity. To strengthen the weakest muscle parts through practice, bimanual activities are performed frequently with increasing difficulty levels. Enhancing ROM and functional ability is targeted using VR (Figure 3) to increase motor performance for 30 minutes, three days a week, with progression based on muscle strength. Promoting personal autonomy involves frequently practicing tasks such as dressing, eating, bathing, brushing teeth, and eventually combing hair, cutting, drawing, writing, and doing puzzles. Finally, nerve regeneration is promoted through FES at 1 mA, as mentioned in Table 4.

**Table 4:** Structured rehabilitation Interventions were carried out for four weeks, six days for one hour ROM: range of motion; FES: functional electrical stimulation; VR: virtual reality

Goal	Intervention	Intensity	Progression
To improve the strength of the affected region	Free exercise regimens, strengthening exercise, multiple angles isometric, brief resisted isometric	20 minutes, five days/week 1	Mechanical resisted exercises and progressive resisted exercise
Improving the utilization of affected extremities	Modified constraint-induced movement therapy	Six hours a day, divided into two with an hour gap between each	An increase in sensorimotor frequency and intensity
To strengthen the weakest muscle parts with practice	Bimanual activity	Frequently	Increasing the difficulty level
To improve ROM and functional ability	VR to increase motor performance (Figure 3B)	30 minutes, three days/week	Progression according to muscle strength
To promote personal autonomy	Dressing oneself, eating, bathing, and brushing the teeth	Frequently	Combing hair, cutting, drawing, writing, and doing puzzles
To promote nerve regeneration	FES (Figure 3A)	Intensity: 10-20 mA, frequency: 20 Hz, pulse duration: 200 µs, duration: 10-15 minutes, on/off ratio: 10 seconds on/20-30 seconds off	-

Outcome measures

Upper extremity (UE) function was assessed using the upper extremity functional index (UEFI) and the brachial plexus outcome measure (BPOM) both before and after the intervention (Table 5). The BPOM scale contains 14 items that measure activity, and the reliability of the self-evaluation test-retest was high (0.938) [14]. UEFI measures how much UE disorders limit an individual's activities. The total score varied from 0 (worst function) to 80 (best function), with the 20 items having a score ranging from 0 (very difficult or unable to perform activity) to 4 (no difficulty). It offers the best test-retest reliability. Results show that following a six-week intervention, the outcome measure significantly improved [15]. The Manual Ability Classification System (MACS) describes how children with cerebral palsy use their hands to handle objects in daily activities. MACS has good validity and reliability. The intraclass correlation coefficient between therapists was 0.97 (95% CI, 0.96-0.98), and between parents and therapist was 0.96 (95% CI, 0.89-0.98), indicating excellent agreement. Functional independence measures for children are measures of functional ability that can be used for typically developing children aged six months to seven years, as well as children over seven years with disabilities and delays in functional development.

**Table 5:** Outcome measure BPOM: brachial plexus outcome measure; UEFI: upper extremity functional index; MACS: Manual Ability Classification System; WeeFIM: functional independence measure

Outcome measure	Preintervention	Postintervention
BPOM	49/55	53/55
UEFI	50/80	65/80
MACS	Level 3	Level 4
WeeFIM	86/126	110/126

## Discussion

Erb's palsy results in significant functional deficits [16] and impairs the UE's daily activities. Traumatic BPI is produced by forceful events that result in the loss of UE functionality. Physiotherapeutic interventions have been shown to improve UE function [17]. This case study aims to demonstrate the efficacy of personalized physiotherapy treatment in increasing upper limb function. The BPOM and UEFI were used as outcome measures to assess the UE function.

Solomen et al. described the rehabilitation of a 23-year-old male with a BPI in their case report. They concluded that after six months of systematic therapy, the AROM was close to normal, and the patient achieved Grade 5 muscle power [18]. In their study, Eren et al. discovered that mCIMT promotes more functional improvements than traditional rehabilitation programs and improves forearm supination and elbow flexion AROMs, forearm supination function, gross motor abilities, and handgrip strength of the afflicted limb [19].

Cavdar et al. [20] concluded in their comprehensive review that, in VR applications, neuroplasticity is the brain's ability to reorganize itself by forming new neural connections throughout life. VR can stimulate neuroplasticity by providing repetitive, engaging tasks that encourage the brain to adapt and learn new skills. Either alone or in combination with physical therapy interventions, these methods are beneficial in improving ROM, upper limb functions, and muscle strength in children with obstetric BPI (OBPI). However, high-quality research, a larger sample size of individuals with OBPI, and an assessment of long-term outcomes are required [11]. The research by Frade et al. describes a case of a child with neonatal brachial plexus palsy on the right arm with damage to the C5, C6, and C7 nerves. The symptoms at birth and diagnosis were the absence of movement in the right arm but finger mobility. The rehabilitation provided the child with a functioning limb for daily activities, including bimanual motor integration and coordination, PROM, and AROM in the various joints except for pronation, sensibility, and maintaining strength. Finally, we may say that this case report describes a set of rehabilitation strategies [21].

Yang et al. concluded that although surgical treatment can improve the function of a damaged brachial plexus, it carries some risk and is rarely the first option. FES is effective in stimulating damaged nerve regeneration and avoiding skeletal muscle denervation atrophy, making it a viable therapy option for BPI [13]. This study shows the effect of VR and FES and shows that when given, VR and FES improve ROM, strength, and functional ability.

## Conclusions

Patients experience challenges in performing daily tasks after traumatic events like Erb's palsy. In this case report, a structured rehabilitation using VR and FES demonstrated a significant improvement in UE function in a seven-year-old girl after four weeks of rehabilitation. Thus, VR and FES can be considered a beneficial intervention to improve the functional ability of the UE after Erb's palsy.

## References

[1] Patra S, Kurup JKN, Acharya AM, Bhat AK (2016). Birth brachial plexus palsy: a race against time. BMJ Case Rep.

[2] Sandmire HF, DeMott RK (2002). Erb's palsy causation: a historical perspective. Birth.

[3] Carlson Strother C, Joslyn-Eastman N, Loosbrok MF, Pulos N, Bishop AT, Spinner RJ, Shin AY (2022). Surgical management of traumatic brachial plexus injuries in the pediatric population. World Neurosurg.

[4] Dorsi MJ, Hsu W, Belzberg AJ (2010). Epidemiology of brachial plexus injury in the pediatric multitrauma population in the United States. J Neurosurg Pediatr.

[5] Doi K, Otsuka K, Okamoto Y, Fujii H, Hattori Y, Baliarsing AS (2002). Cervical nerve root avulsion in brachial plexus injuries: magnetic resonance imaging classification and comparison with myelography and computerized tomography myelography. J Neurosurg.

[6] Szaro P, Geijer M, Ciszek B, McGrath A (2022). Magnetic resonance imaging of the brachial plexus. Part 2: traumatic injuries. Eur J Radiol Open.

[7] Milano MT, Mavroidis P, Ryckman J (2023). Radiation-induced inferior brachial plexopathy after stereotactic body radiotherapy: pooled analyses of risks. Radiother Oncol.

[8] Simmons Z (2013). Electrodiagnosis of brachial plexopathies and proximal upper extremity neuropathies. Phys Med Rehabil Clin N Am.

[9] Raducha JE, Cohen B, Blood T, Katarincic J (2017). A review of brachial plexus birth palsy: injury and rehabilitation. R I Med J (2013).

[10] Srilakshmi D, Chaganti S (2013). A holistic approach to the management of Erb's palsy. J Ayurveda Integr Med.

[11] Naqvi WM, Naqvi I, Mishra GV, Vardhan V (2024). The dual importance of virtual reality usability in rehabilitation: a focus on therapists and patients. Cureus.

[12] Bryanton C, Bossé J, Brien M, McLean J, McCormick A, Sveistrup H (2006). Feasibility, motivation, and selective motor control: virtual reality compared to conventional home exercise in children with cerebral palsy. Cyberpsychol Behav.

[13] Rich JA, Newell A, Williams T (2019). Traumatic brachial plexus injury rehabilitation using neuromuscular electrical muscle stimulation in a polytrauma patient. BMJ Case Rep.

[14] Hosbay Z, Ozkan S, Tanriverdi M, Aydin A (2019). Reliability and validity of the brachial plexus outcome measure in children with obstetric brachial plexus palsy. J Hand Ther.

[15] Gabel CP, Michener LA, Burkett B, Neller A (2006). The upper limb functional index: development and determination of reliability, validity, and responsiveness. J Hand Ther.

[16] Shah V, Coroneos CJ, Ng E (2021). The evaluation and management of neonatal brachial plexus palsy. Paediatr Child Health.

[17] Srushti Sudhir C, Sharath HV (2023). A brief overview of recent pediatric physical therapy practices and their importance. Cureus.

[18] Solomen S, Babu B, Muralidharan PC, Sreejith K, Gafoor A (2021). Conservative management of brachial plexus injury through a structured rehabilitation protocol: a case report. RGUHS J Physiother.

[19] Eren B, Karadağ Saygı E, Tokgöz D, Akdeniz Leblebicier M (2020). Modified constraint-induced movement therapy during hospitalization in children with perinatal brachial plexus palsy: a randomized controlled trial. J Hand Ther.

[20] Cavdar FA, Demir TB, Uz ZL, Ayhan Ö, Mustafaoglu R (2023). Effects of virtual reality applications on children with obstetric brachial plexus injury: a systematic review. Int J Basic Clin Stud.

[21] Frade F, Neves L, Florindo-Silva F, Gómez-Salgado J, Jacobsohn L, Frade J (2022). Rehabilitation of a child with neonatal brachial plexus palsy: case report described by parents. Children (Basel).

